# Personal

**Published:** 1897-07

**Authors:** 


### Personal.

Dr. N. R. Coleman, of Columbus, O., after a continuous service of about eighteen years as an instructor in medical colleges, has retired, at least temporarily, from college work. For a number of years he was Professor of Physical Diagnosis, and for one year Professor of Diseases of Women and Children in Columbus Medical College. From the founding of the Ohio Medical University he has filled the chair of theory and practice of medicine and clinical medicine, with acceptance and distinguished ability. His untiring efforts in behalf of medical legislation, his arduous duties as president of the State Board of Medical Registration and Examination, added to those of his professorship in the university, and membership on the Board of Examining Surgeons for Pensions, and with the care of a large general practice, have been for some time a great strain upon his health, and have made curtailment of professional work a necessity. The friends of medical education will regret that curtailment of work should not have been made in some other direction. Dr. Coleman has rendered the profession at large signal service in the efficient discharge of professional and public duties, and he retires from the faculty of Ohio medical university with the good will and best wishes of all his colleagues in that institution.

We trust that his health may long be spared and that he and his associates on the state board may carry forward the good work which they have so well begun of elevating the medical practice and the educational requirements of the medical colleges of the state.—*Columbus Med. Jour.*

---

Dr. Henry D. Didama, of Syracuse, having reached the fiftieth year of his medical practice, was given a dinner in commemoration of the event by the faculty and the alumni association of the college of medicine, Syracuse University, Wednesday evening, June 9, 1897. Dr. Nathan Jacobson presided as toastmaster and among the speakers announced were Dr. Albert Vander Veer, of Albany, whose subject was His Alma Mater; Dr. Matthew D. Mann, of Buffalo, subject, Medical education, and Dr. William S. Ely, of Rochester, whose subject was The state board of medical examiners.

---

Dr. Frederick C. Peterson, junior house surgeon at the Fitch Accident Hospital, has been appointed interne at the lying-in department of the New York Foundling Hospital. Dr. Peterson is an alumnus of the University of Buffalo, class of '96. His home is at Watertown.

---

Dr. John B. Hamilton, editor of the *Journal of the American Medical Association*, late surgeon of the Marine Hospital Service, has been elected superintendent of the Illinois Northern Hospital for the Insane at Elgin.

---

DR. MILO B. WARD, of Topeka, Kan., has decided to locate in Kansas City, Mo., and will occupy offices in the Rialto. Dr. Ward has long occupied an enviable position as gynecologist and abdominal surgeon in Kansas. He will take high rank in Kansas City from the first. We welcome the doctor to *the* medical center of the West.—*Langsdale's Lancet.*

---

DR. HENRY OSTHUES, late of 1266 Jefferson street, Buffalo, has removed to 769 Quincy street, Brooklyn, N. Y. Dr. Osthues was formerly demonstrator of anatomy in the medical department Niagara University and secretary of the alumni association. His many friends in Buffalo will be glad to learn of his successful beginning in his new location.

---

DR. W. A. ADAMS, of Fort Worth, Texas, chief surgeon of the Fort Worth and Denver City railway company, has lately been the recipient of the degree of LL. D. from Mercer University. The compliment is a well-bestowed honor and Mercer University will not suffer thereby.

